# Understanding digital diffusion and online decision-making in public health promotion: a sequential mixed-methods study of the violence prevention charter in Canada

**DOI:** 10.3389/fpubh.2026.1876403

**Published:** 2026-09-24

**Authors:** Shannon Turner

**Affiliations:** School of Health and Behavioural Sciences, Torrens University Australia, Melbourne, VIC, Australia

**Keywords:** complex adaptive systems, diffusion of innovations, digital public health, health promotion, mixed methods, online decision-making, social network analysis, violence prevention

## Abstract

Digital environments have become important settings for public health promotion, yet dissemination alone does not explain why individuals and organizations choose to adopt and act on public health information. Understanding digital diffusion requires attention to temporal patterns of adoption, the social networks through which information and influence circulate, and the contextual conditions that shape decision-making. This sequential mixed-methods single case study examined the dissemination and voluntary adoption of the Violence Prevention Charter, a principles-based public health initiative in Canada. Website analytics and Bass diffusion modeling characterized temporal patterns of dissemination and adoption. Social Network Analysis examined the relational environment through which information, trust, and influence circulated, while a realist-informed examination of Context–Mechanism–Outcome configurations explored the contextual conditions and generative mechanisms influencing participant reasoning and engagement. The complementary findings were integrated and interpreted through a Complex Adaptive Systems framework. Digital engagement demonstrated sustained dissemination and an accelerating pattern of adoption following purposeful promotion. Social Network Analysis identified a cohesive professional network characterized by strong trust relationships, information exchange, and influential connections. Realist-informed analysis identified three principal mechanisms influencing engagement: creation of social good, trust in the source, and influence of colleagues and peers. Adoption decisions were also shaped by competing demands, characteristics of the digital environment, organizational context, and participants’ independent assessment of the relevance and credibility of the initiative. The findings suggest that diffusion within digital public health ecosystems is shaped by interactions among communication, trusted social relationships, organizational context, and individual agency. Sequential integration of temporal, relational, and contextual methods provides a useful approach for investigating online decision-making and voluntary adoption, while a Complex Adaptive Systems perspective offers a framework for understanding how these interacting influences contribute to the diffusion of principles-based public health interventions.

## 1 Introduction

Violence is a significant and persistent public health issue with profound consequences for individuals, families, communities, and health systems worldwide. Public health approaches to violence prevention have increasingly emphasized upstream, population-based strategies that address the social, structural, and environmental determinants of violence alongside coordinated, multisectoral action. While substantial progress has been made in developing evidence-informed policies and interventions, less attention has been directed toward understanding how public health knowledge, principles, and frameworks themselves are disseminated, interpreted, and voluntarily adopted across complex organizational and community settings.

The rapid expansion of digital communication has fundamentally altered how public health information is created, shared, and acted upon. Digital environments now function as important settings for health promotion, enabling information to move across professional, organizational, and community boundaries through dynamic many-to-many communication rather than traditional linear dissemination pathways. Within these environments, adoption is influenced not only by the characteristics of the information itself, but also by the social networks through which it travels, the relationships that support trust and credibility, and the contextual conditions that shape how individuals and organizations interpret and apply new ideas. Understanding these interconnected processes has become increasingly important as digital platforms assume a central role in public health communication and knowledge mobilization.

The Violence Prevention Charter (VPC) was developed as a voluntary, principles-based public health initiative intended to encourage organizations and individuals to publicly endorse a shared commitment to violence prevention. Rather than prescribing specific actions or implementation requirements, the Charter invited participants to adopt a common set of guiding principles that could be interpreted and operationalized within their own organizational and community contexts. This characteristic made the VPC particularly well suited for examining how a public health intervention diffuses and is voluntarily adopted within a digital ecosystem, where dissemination, interpretation, and engagement emerge through interactions among individuals, organizations, and communities rather than through centralized implementation.

Research examining the diffusion of innovations has long recognized that adoption is influenced by communication, social influence, and the characteristics of both innovations and adopters (1, 2). More recently, digital public health has expanded these perspectives by recognizing digital environments as complex sociotechnical systems in which information flows, social interaction, and contextual influences continually shape engagement and decision-making. While these perspectives have advanced understanding of digital communication, relatively few studies have examined how complementary analytical approaches can be deliberately integrated to investigate the temporal, relational, and contextual dimensions of the same public health intervention within a single coherent investigative framework.

This study addresses that gap through an in-depth single case study of the Violence Prevention Charter. A sequential mixed-methods design was employed to investigate complementary dimensions of the Charter’s dissemination and adoption within a digital ecosystem. Website analytics and diffusion modeling characterized temporal patterns of dissemination and adoption. Social Network Analysis (SNA) characterized the relational environment through which information, trust, and influence circulated. A realist-informed examination of Context–Mechanism–Outcome (CMO) configurations explored the contextual mechanisms influencing participant reasoning and engagement. Each methodological component was conducted as an analytically independent line of inquiry while contributing complementary evidence to an integrated understanding of the same adoption process.

The study was designed as an iterative investigative sequence in which findings from each analytical perspective informed subsequent inquiry, progressively developing a more comprehensive understanding of the case. Rather than relying on a single methodological perspective, complementary lines of evidence were deliberately integrated to examine how temporal patterns, social relationships, and contextual mechanisms interacted to influence the voluntary diffusion and adoption of the Charter. Complex Adaptive Systems (CAS) provided the overarching explanatory framework through which these integrated findings were interpreted, recognizing that public health interventions become embedded within dynamic systems where adaptation emerges through interactions among information, people, organizations, and context.

The purpose of this study was not to develop a universally generalizable model of diffusion and adoption, but to undertake a rigorous examination of one carefully bounded case to better understand how a principles-based public health intervention diffused through a digital ecosystem. In doing so, the study contributes to growing scholarship at the intersection of public health, digital health, implementation science, and information science by illustrating how complementary analytical perspectives can be sequentially integrated to investigate complex processes of information dissemination, interpretation, and voluntary adoption.

## 2 Materials and methods

### 2.1 Study design

This study employed a sequential mixed-methods single case study design to investigate the diffusion and adoption of the Violence Prevention Charter (VPC) within a digital ecosystem. The study sought to better understand how a principles-based public health intervention was disseminated, interpreted, and voluntarily adopted by examining complementary temporal, relational, and contextual dimensions of the same adoption process. Recognizing that no single methodological approach could adequately explain these interconnected dimensions, a pragmatic mixed-methods design was adopted in which analytically independent methods were deliberately selected to address distinct but complementary research questions (3–5).

The Violence Prevention Charter was investigated as a bounded single case because it provided an opportunity to examine the dissemination and voluntary adoption of a principles-based public health intervention within its natural digital environment. Rather than evaluating implementation of a prescribed intervention, the study examined how individuals and organizations interpreted, shared, and adopted common principles across diverse organizational and community contexts. A single case study design enabled an in-depth examination of this bounded phenomenon while preserving the contextual relationships necessary to understand a complex public health intervention (6–8).

The methodological design was conceived as an iterative investigative sequence in which each analytical component addressed a distinct but complementary dimension of the same adoption process. Website analytics and diffusion modeling characterized temporal patterns of dissemination and adoption. Social Network Analysis (SNA) characterized the relational environment through which information, influence, trust, and shared principles circulated. A realist-informed examination of Context–Mechanism–Outcome (CMO) configurations explored the contextual mechanisms influencing participant reasoning and decisions to engage with the Charter. Although each methodological component was conducted as an analytically independent line of inquiry, the sequential design enabled complementary lines of evidence to iteratively inform subsequent analyses and progressively enrich interpretation. Understanding therefore emerged through the deliberate integration of complementary evidence rather than through reliance on any single analytical perspective alone (9–11).

This investigative sequence was designed to progressively reduce uncertainty surrounding the observed adoption process. Each analytical perspective addressed a different source of uncertainty while informing the interpretation and refinement of subsequent analyses. Temporal analyses established when dissemination and adoption occurred; relational analyses characterized the network structures within which these processes unfolded; and realist-informed inquiry examined the contextual mechanisms influencing participant engagement. Together, these complementary perspectives provided an increasingly comprehensive understanding of the same adaptive process while preserving the methodological integrity of each analytical approach.

Complex Adaptive Systems (CAS) provided the overarching explanatory framework for interpreting the integrated findings rather than functioning as an analytical method. From a CAS perspective, public health interventions become embedded within the systems they seek to influence, where interactions among individuals, organizations, information, and context generate adaptive responses that cannot be adequately understood through isolated analyses alone (12–15). Accordingly, the objective of the sequential mixed-methods design was not to explain complexity through any individual method, but to develop an increasingly comprehensive understanding of how temporal patterns, social relationships, and contextual mechanisms interacted within a digital ecosystem to influence the diffusion and adoption of the Violence Prevention Charter.

### 2.2 Study context

The Violence Prevention Charter (VPC) was developed by the Violence Prevention Community (VPC), a voluntary, interdisciplinary network committed to advancing violence prevention through collaboration, knowledge exchange, and collective action. The Charter articulates a shared set of evidence-informed principles that individuals and organizations may voluntarily endorse as a public declaration of their commitment to violence prevention. Unlike interventions that prescribe standardized implementation activities, the Charter was intentionally designed as a flexible, principles-based initiative that could be interpreted and operationalized within diverse organizational and community contexts.

This characteristic made the Charter an appropriate case through which to investigate the diffusion and voluntary adoption of a public health intervention within a digital ecosystem. Adoption did not require organizational mandates or prescribed implementation pathways. Rather, individuals and organizations independently engaged with the Charter, interpreted its principles within their own contexts, and determined whether and how to adopt and promote its commitments. Consequently, dissemination and adoption emerged through interactions among communication processes, social relationships, organizational contexts, and participant decision-making rather than through centralized implementation.

The study therefore examined the Charter as a bounded case of voluntary public health innovation operating within a naturally occurring digital environment. The focus was not the effectiveness of the Charter itself, but the processes through which information about the Charter was disseminated, interpreted, and adopted across an interconnected network of public health practitioners, organizations, and community stakeholders.

### 2.3 Investigative sequence

The investigative sequence was deliberately structured so that each methodological component examined a distinct but complementary dimension of the same adoption process while informing subsequent inquiry. Rather than applying multiple methods to independently investigate separate research questions, the study employed analytically independent methods that generated complementary lines of evidence addressing when dissemination occurred, how information and shared principles moved through social networks, and why individuals and organizations chose to engage under particular contextual conditions.

The first phase established the temporal characteristics of dissemination using website analytics and diffusion modeling. These analyses identified patterns of initiation, acceleration, and stabilization that provided the empirical foundation for subsequent investigation. The second phase employed Social Network Analysis to characterize the relational environment through which information, trust, and influence circulated. Rather than mapping diffusion to individual participants, the analysis examined network cohesion, centrality, connectivity, and bridging relationships that shaped opportunities for information exchange and adoption. The third phase used a realist-informed examination of Context–Mechanism–Outcome configurations to explain how contextual conditions influenced participant reasoning and decisions to engage with the Charter. Finally, complementary findings from each analytical perspective were integrated and interpreted through the overarching Complex Adaptive Systems framework to develop a systems-informed understanding of the diffusion and adoption process.

This investigative sequence reflects contemporary mixed-methods approaches in which analytically independent methods contribute complementary lines of evidence that are deliberately integrated to develop a richer understanding of complex phenomena than could be achieved through any single analytical perspective alone (3, 9, 11).

### 2.4 Digital engagement analytics and diffusion modeling

The first phase of the investigation sought to characterize the temporal dynamics of dissemination and adoption associated with the Violence Prevention Charter (VPC). Establishing when engagement occurred, how it evolved over time, and whether recognizable patterns of diffusion emerged was essential for providing the empirical foundation upon which the subsequent relational and contextual analyses could build. Google Analytics was therefore used to characterize patterns of online engagement, while diffusion modeling provided an interpretive framework for understanding the observed adoption trajectory.

Google Analytics (Universal Analytics) was used throughout the study period to monitor user engagement with the Violence Prevention Charter website. Metrics including website visits, page views, referral pathways, geographic distribution of users, and patterns of interaction were examined to characterize dissemination over time. These measures provided an objective record of engagement activity and enabled temporal patterns associated with dissemination and adoption to be identified. Within digital public health, website analytics are increasingly recognized as valuable indicators of information dissemination, user engagement, and knowledge mobilization within digitally connected health systems (16, 17).

To further examine the observed temporal patterns, the Bass Diffusion Model (1) was applied as an interpretive framework. The model distinguishes adoption arising through external influences (innovation) from adoption resulting from interpersonal communication and social influence (imitation), providing a well-established theoretical framework for understanding the diffusion of innovations over time. Although originally developed to describe consumer innovation adoption, the Bass model has subsequently informed research examining the diffusion of innovations across healthcare and public health organizations (1, 2, 18).

The Bass Diffusion Model is represented as:


$$\frac{f(t)}{1–F(t)}=p+\mathrm{qF}(t)$$

where:

- f(t) represents the probability density function describing adoption at time *t*;
- F(t) represents the cumulative proportion of adopters by time *t*;
- p represents the coefficient of innovation, reflecting adoption resulting primarily from external influences;
- q represents the coefficient of imitation, reflecting adoption influenced by interpersonal communication and social interaction.

Within this study, the Bass model was used as an interpretive framework to characterize the observed dissemination trajectory rather than as a predictive model. Application of the model enabled the observed website activity to be interpreted in relation to established innovation diffusion theory while providing the temporal foundation for the subsequent relational and contextual analyses. The resulting diffusion curve and model interpretation are presented in the Results.

Although the Bass model provides a robust theoretical framework for characterizing innovation diffusion, contemporary digital health scholarship recognizes that adoption within health systems is influenced by a broader range of organizational, technological, and contextual factors than can be explained through temporal diffusion patterns alone. Frameworks such as the Non-adoption, Abandonment, Scale-up, Spread, and Sustainability (NASSS) framework emphasize that adoption emerges through complex interactions among technologies, individuals, organizations, and wider system contexts rather than through linear diffusion processes alone (19). Consistent with this broader perspective, the Bass model was not used in isolation, but as one component of a sequential mixed-methods investigation in which temporal diffusion patterns were subsequently examined alongside relational and contextual evidence to develop a more comprehensive understanding of the observed adoption process.

While the temporal analyses established when dissemination and adoption occurred, they could not explain how information and shared principles moved through the community of adopters or why individuals and organizations chose to engage. Understanding these relational dynamics required examination of the underlying social network through which information, influence, and trust circulated, providing the rationale for the subsequent Social Network Analysis.

### 2.5 Social Network Analysis

Following characterization of the temporal diffusion pattern, the second phase of the investigation examined the relational environment within which dissemination occurred. While digital engagement analytics established when dissemination and adoption occurred, they could not explain how information, influence, and shared principles circulated among individuals and organizations. Social Network Analysis (SNA) was therefore undertaken to characterize the structure and relationships of the Violence Prevention Community (VPC) network and to examine the social environment through which dissemination occurred.

Social Network Analysis is a well-established methodological approach for examining patterns of relationships among individuals and organizations and understanding how network structure influences communication, collaboration, knowledge exchange, and the diffusion of innovations (20–22). Within public health and implementation science, SNA has increasingly been applied to investigate collaborative networks, knowledge translation, implementation processes, and the dissemination of evidence-informed practices across professional and organizational systems (23–25).

Consistent with contemporary implementation and knowledge translation research, Social Network Analysis was not undertaken as a stand-alone investigation but as one complementary analytical perspective within an iterative mixed-methods investigative sequence. Its purpose was to characterize the relational environment within which information exchange, collaboration, and knowledge mobilization occurred, thereby providing the network context required to interpret the temporal diffusion patterns identified in the preceding phase (23).

Network data were collected to characterize relationships among members of the Violence Prevention Community and participating organizations. Network visualizations and structural measures were used to examine characteristics including network cohesion, density, connectivity, centrality, and bridging relationships. Particular attention was given to these structural properties because they influence opportunities for information exchange, collaboration, knowledge mobilization, and the diffusion of innovations across organizational systems (20, 21, 23).

Importantly, the purpose of the Social Network Analysis was not to trace individual pathways of adoption or establish causal mechanisms of diffusion. Rather, the analysis characterized the relational environment within which dissemination occurred, providing insight into the structural opportunities through which information, influence, and shared principles could circulate. These findings complemented the temporal analyses by describing the network context in which the observed diffusion pattern emerged, while recognizing that network structure alone cannot explain participant decision-making or adoption.

Within the investigative sequence, Social Network Analysis served as the bridge between temporal diffusion and contextual explanation. Having established when dissemination occurred, the analysis provided insight into how the relational structure of the network supported communication, trust, and knowledge exchange. However, understanding why individuals and organizations chose to engage with the Violence Prevention Charter required examination of the contextual mechanisms influencing participant reasoning and decision-making. This provided the rationale for the subsequent realist-informed inquiry.

### 2.6 Integration of complementary lines of evidence

The analytical objective of this study was not to compare independent datasets, but to develop an increasingly comprehensive understanding of the diffusion and voluntary adoption of the Violence Prevention Charter through the deliberate integration of complementary lines of evidence. Each methodological component remained analytically independent while contributing a distinct analytical perspective on the same bounded case. Consequently, integration occurred throughout the investigative sequence rather than solely at its conclusion, with findings from each phase informing the interpretation and refinement of subsequent analyses.

This sequential integration was guided by the principle that no single methodological approach could adequately explain the processes influencing the diffusion and voluntary adoption of the Violence Prevention Charter. Rather than viewing the temporal, relational, and contextual analyses as separate investigations, the study treated them as complementary analytical perspectives examining different aspects of the same case. Each analytical perspective addressed a distinct research question while contributing to a progressively more complete understanding of the diffusion and adoption process.

Digital engagement analytics established when dissemination and adoption occurred by characterizing temporal patterns of engagement. Social Network Analysis subsequently examined how information, influence, trust, and shared principles circulated through the relational environment. Finally, the realist-informed examination of Context–Mechanism–Outcome (CMO) configurations explored why individuals and organizations chose to engage with, endorse, and promote the Charter within particular organizational and community contexts.

The integration of these complementary analytical perspectives enabled interpretation to develop progressively throughout the investigation. Findings from each analytical phase informed the interpretation of subsequent analyses while preserving the methodological integrity and independent contribution of each approach. Temporal analyses established the chronology of dissemination and adoption, Social Network Analysis characterized the relational environment within which communication and knowledge exchange occurred, and the realist-informed inquiry explained the contextual mechanisms influencing participant reasoning and decision-making. Together, these complementary lines of evidence provided a more comprehensive understanding of the diffusion and voluntary adoption of the Violence Prevention Charter than could have been achieved through any single analytical approach alone (9–11, 26).

Importantly, integration was interpretive rather than confirmatory. The objective was not to seek agreement across datasets or to validate one methodological perspective against another. Instead, each analytical component contributed unique explanatory insight that complemented and extended the findings of the preceding phases. Differences among the findings were not viewed as inconsistencies requiring reconciliation but as complementary perspectives that enriched understanding of the case. This approach is consistent with contemporary mixed-methods research, which recognizes that integrating diverse forms of evidence can provide a more complete understanding of complex research questions while preserving the integrity of individual methodological approaches (3, 9).

The integrated interpretation developed through this investigative sequence subsequently provided the foundation for examination through the Complex Adaptive Systems (CAS) framework. Rather than functioning as an analytical method, CAS served as an explanatory framework through which the interactions among temporal diffusion, network relationships, participant reasoning, and organizational context could be understood collectively. This final stage of interpretation enabled the findings from the individual analytical components to be considered together as parts of a dynamic public health system, providing a systems-informed understanding of the processes influencing the diffusion and voluntary adoption of the Violence Prevention Charter.

### 2.7 Ethics

Ethical approval for this study was obtained from the University of Victoria Human Research Ethics Board in accordance with the Tri-Council Policy Statement: Ethical Conduct for Research Involving Humans (TCPS) and the ethical principles of the Declaration of Helsinki. Following the transfer of the doctoral candidature to Torrens University Australia, the approved ethics protocol was reviewed and accepted by the University as part of the candidate’s continuing research registration and governance requirements.

All participants received written information describing the purpose of the study, the voluntary nature of participation, and the procedures for informed consent prior to participation. Separate informed consent procedures were implemented for participation in the Violence Prevention Charter, the qualitative interviews and focus groups, and the Social Network Analysis. Participation was voluntary, and participants were advised that they could decline to answer any question or withdraw from the study at any time without consequence.

The study incorporated digital engagement analytics, Social Network Analysis, and qualitative interviews and focus groups. Website analytics were analyzed only in aggregate and did not involve the identification of individual users. Social Network Analysis data were anonymized prior to analysis using unique participant identifiers, and identifiable information was accessible only to the research team for the purposes of data preparation. Interview and focus group transcripts were de-identified before analysis, and all electronic data were securely stored on password-protected systems in accordance with the approved ethics protocol.

Throughout the investigation, care was taken to protect participant confidentiality while preserving the analytical integrity of each methodological component. The integration of complementary lines of evidence was undertaken only after each analytical phase had been completed independently, ensuring that each analytical approach retained its methodological integrity while contributing to the integrated interpretation of the case.

## 3 Digital engagement analytics and diffusion modeling

### 3.1 Digital engagement

Digital engagement analytics and diffusion modeling characterized the temporal pattern of dissemination and voluntary adoption of the Violence Prevention Charter (VPC) over the study period. Collectively, these analyses established the chronology of adoption and provided the empirical foundation for the subsequent examination of network relationships and contextual mechanisms.

Website analytics recorded 1,548 user visits and 4,026 page views between January 1, 2017, and July 11, 2019, demonstrating sustained engagement with the Charter throughout the dissemination period. The landing page received 1,943 unique page views, while the “Sign the Charter” page received 546 page views, indicating that a substantial proportion of users progressed beyond initial awareness to the point of potential endorsement. Website visitors reached the Charter through multiple referral pathways, including direct access, organizational websites, search engines, professional communications, and social media, demonstrating that dissemination occurred through multiple complementary channels rather than a single source. Most users accessed the website via desktop computers (1,281 users), compared with 224 mobile and 43 tablet users, while 83% of website activity occurred through the English-language pages.

The cumulative pattern of Charter adoption demonstrated the characteristic S-shaped diffusion trajectory described by the Bass Diffusion Model (1). Initial adoption was gradual and corresponded with early dissemination activities and direct promotion of the Charter. This period was followed by a sustained phase of accelerating adoption, during which endorsement increasingly occurred through professional relationships, organizational communication, and peer influence. As adoption matured, the rate of new endorsements gradually declined, consistent with a slowing rate of diffusion as the population of potential adopters became increasingly saturated.

#### 3.1.1 Temporal diffusion of voluntary adoption of the violence prevention charter

Observed cumulative Charter endorsements over the study period with fitted Bass Diffusion Model illustrating the temporal pattern of voluntary adoption (Figure 1).

**Figure 1:**
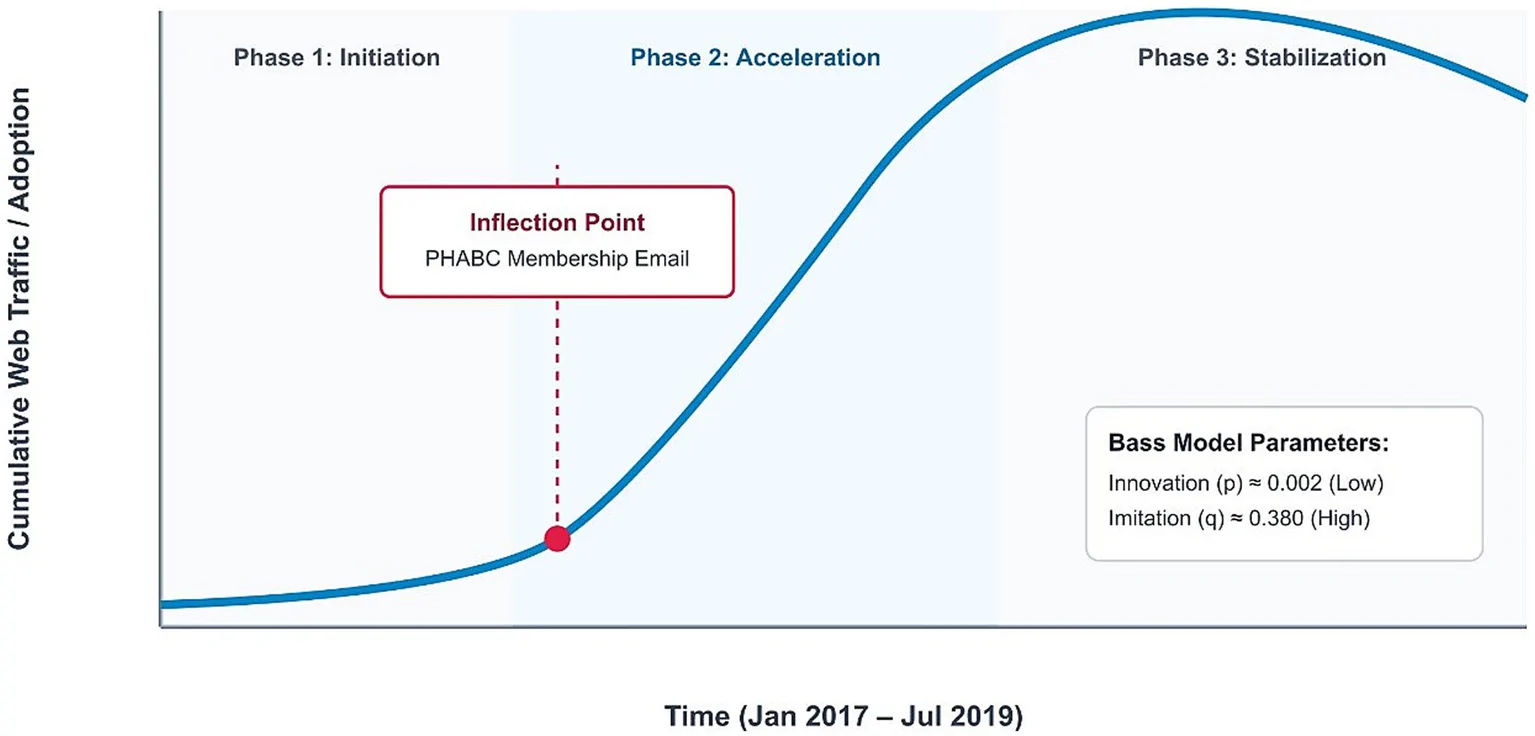
VPC diffusion trajectory bass model curve.

Behavior flow analyses further demonstrated that website users progressed through the site in a logical sequence, with navigation patterns consistently directing users from introductory information toward the Charter endorsement pages. These patterns suggest that the website functioned not only as an information resource but also as a mechanism supporting movement from awareness to active engagement with the Charter.

Together, the digital engagement analytics and diffusion modeling established when dissemination and voluntary adoption occurred and demonstrated that engagement was sustained throughout the study period. While these analyses characterized the temporal pattern of adoption, they could not explain how information, influence, and trust circulated among participants or why individuals and organizations chose to endorse the Charter. These questions were examined through the subsequent Social Network Analysis.

### 3.2 Social Network Analysis

Social Network Analysis characterized the relational structure of the Public Health Association of British Columbia (PHABC), one of the partner organizations participating in the Violence Prevention Charter initiative. The PHABC network served as the bounded organizational network for this component of the investigation. At the time of the survey, PHABC comprised 135 registered members, including approximately 50 members actively participating in committee work, communications, and advocacy activities. A total of 71 members participated in the Social Network Analysis survey, representing approximately 53% of the registered membership. The resulting network comprised 77 nodes connected by 111 relational ties, providing the basis for examining patterns of connectedness, trust, and influence associated with dissemination and voluntary adoption of the Violence Prevention Charter (20, 27).

The survey demonstrated a highly engaged professional network. Seventy percent of PHABC members signed the Violence Prevention Charter following the invitation to participate, while respondents who had not signed the Charter nevertheless completed the Social Network Analysis survey, providing important insight into factors influencing adoption decisions. Although more than half of respondents reported interacting with their contacts less than once per month, almost all respondents reported sharing information across the network, with nearly three-quarters indicating that they both exchanged information and undertook activities together. Initial relationships were most commonly established through colleagues or peers, consistent with PHABC’s role as a professional association.

The baseline trust network among participating organizations is presented in Figure 2. Directed ties represent reported trust relationships between organizations, while node size is proportional to closeness centrality, a structural measure of how readily an organization can reach, or be reached by, other organizations through the shortest paths within the network. Larger nodes therefore occupy more structurally accessible positions within the trust network and may facilitate more efficient communication across the network.

**Figure 2:**
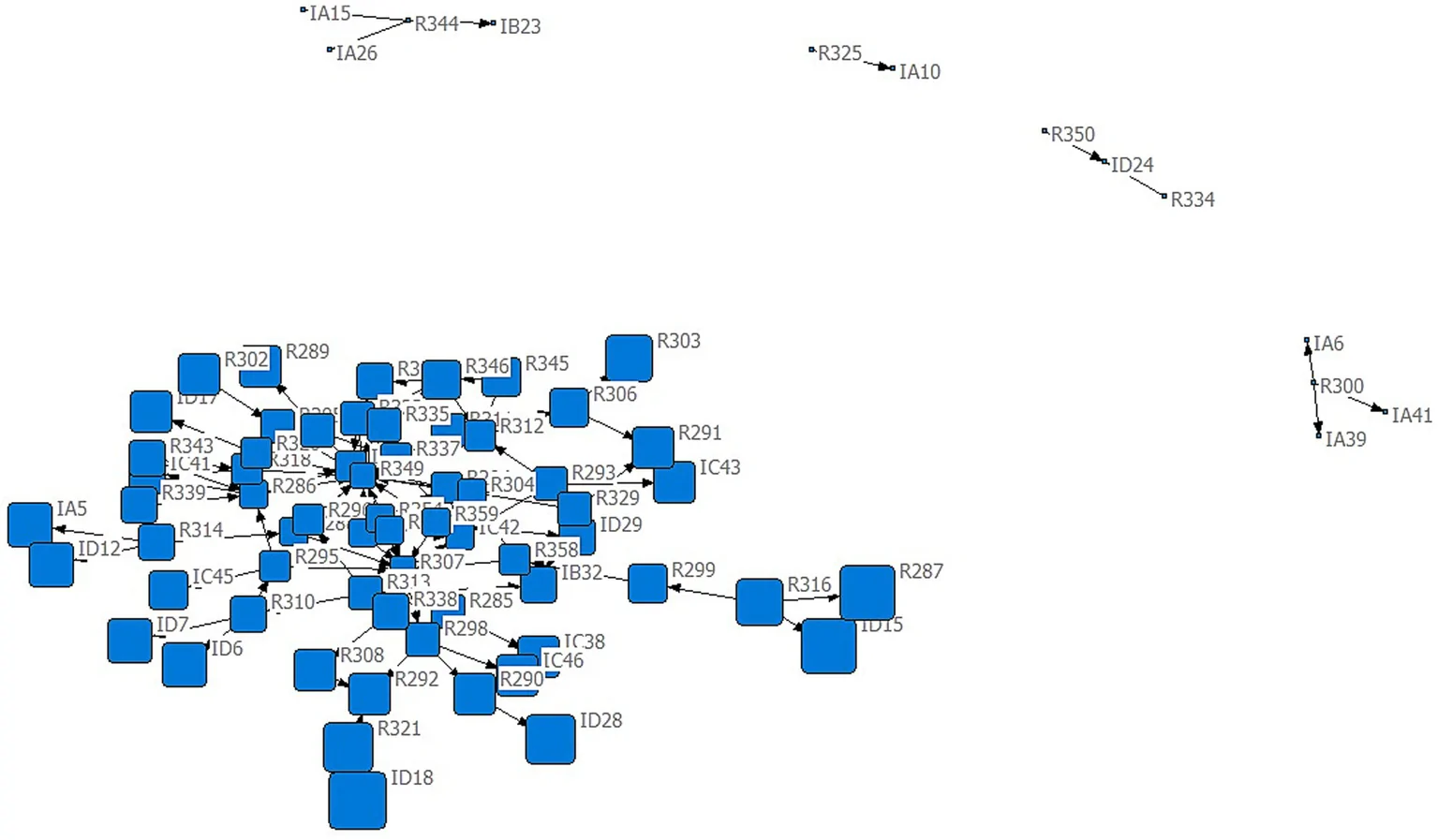
Level of trust by closeness. From results of Violence Prevention Information Sharing Network Analysis Survey, created via UCINET by Analytic Technologies, https://analytictech.com.

The network demonstrates a dense and highly connected core in which most participating organizations are directly or indirectly connected, together with several smaller peripheral components. The overall structure is characterized by high levels of density, reciprocity, and clustering, indicating that participating organizations were embedded within a cohesive professional network of trusted relationships. This structural configuration provided a strong relational foundation for communication and collaboration across the network.

Importantly, closeness centrality describes an organization’s structural position rather than the strength or quality of trust itself. Trust was measured directly through participants’ reported trust relationships, whereas closeness centrality identifies organizations positioned to communicate efficiently with others across the network. Interpreting centrality within a network defined by trust relationships therefore provides insight into how trusted organizational connections may have supported dissemination of the Violence Prevention Charter.

#### 3.2.1 Baseline trust network among participating organizations

Network visualization illustrating the relational structure and relative centrality of members within the PHABC network. Node size reflects relative centrality. Individual identities have been anonymized.

Directed ties represent reported trust relationships among participating organizations. Node size is proportional to closeness centrality, indicating the relative structural accessibility of each organization within the network. Individual organizations have been anonymized. Figures 2, 3 generated using UCINET (Analytic Technologies) from the Violence Prevention Information Sharing Network Analysis Survey.

**Figure 3:**
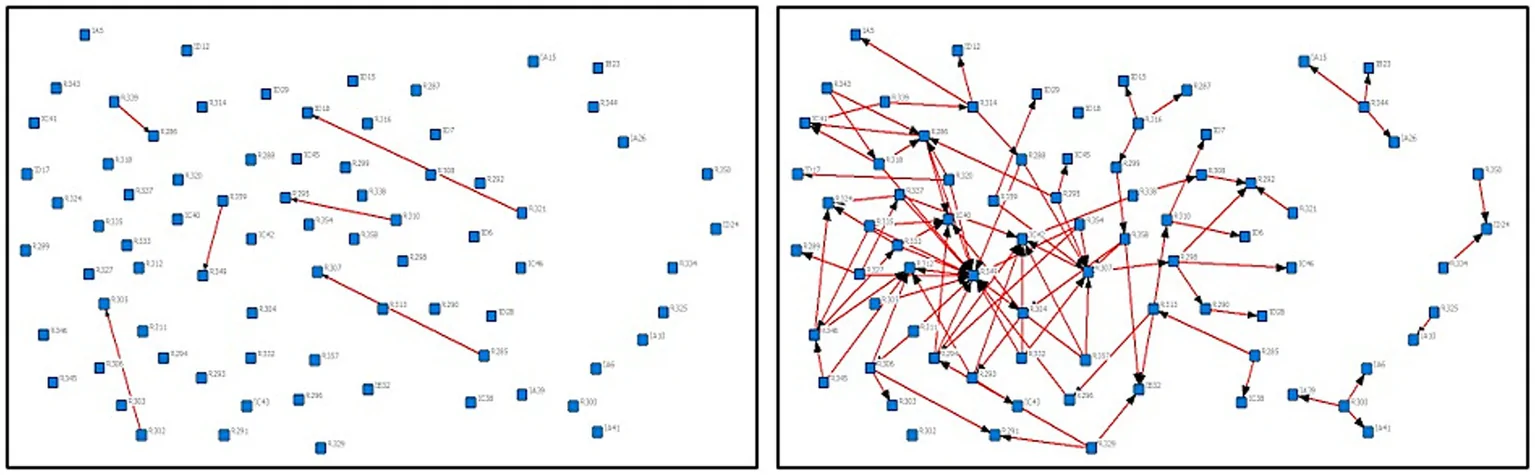
Level of trust networks with directional ties, left to right: Moderate levels of trust, high and very high levels of trust. From results of Violence Prevention Information Sharing Network Analysis Survey, created via UCINET by Analytic Technologies, https://analytictech.com/.

Having established the structural characteristics of the baseline trust network, subsequent analyses examined the reported strength of trust relationships across the PHABC network. These findings are presented in Figure 3.

#### 3.2.2 Strength of trust relationships within the PHABC network

Network visualization illustrating trust relationships among members of the PHABC network. Individual identities have been anonymized.

Level of trust networks with directional ties illustrating moderate, high, and very high reported trust relationships among participating organizations. Individual organizations have been anonymized. Figures 2, 3 generated using UCINET (Analytic Technologies) from the Violence Prevention Information Sharing Network Analysis Survey.

The assessment of reported trust addressed the central question of whether information shared through the network was considered credible by participants. The findings demonstrated overwhelmingly high levels of trust among network members, with respondents reporting high or very high confidence in information shared by their professional contacts. Similarly, respondents indicated that these professional relationships influenced decisions regarding adoption of the Violence Prevention Charter.

Taken together, Figures 2, 3 demonstrate that dissemination occurred within an established professional network characterized by both structural connectedness and strong trust relationships. Figure 2 illustrates the structural organization of the network through closeness centrality, whereas Figure 3 demonstrates the strength of trust reported among participating organizations. Together, these complementary analyses suggest that dissemination occurred through a cohesive and trusted professional network capable of supporting knowledge exchange and voluntary adoption of the Violence Prevention Charter (28).

While the Social Network Analysis characterized the relational structure of the PHABC network and identified the organizational conditions supporting dissemination, it did not explain the contextual reasoning underlying participants’ decisions to engage with or decline the Violence Prevention Charter. These questions were explored through the realist-informed qualitative inquiry presented in the following section.

Network visualization demonstrated a cohesive organizational structure in which information could move across both highly connected members and broader professional relationships. The network contained several more centrally connected members together with bridging relationships linking different areas of the organization. Consistent with Social Network Analysis theory, weaker ties and structural holes contributed to information flow across the network rather than limiting communication to tightly connected clusters (29, 30). The network statistics describing cohesion, density, closeness, reciprocity, and betweenness provided complementary evidence of the organizational structure supporting dissemination of the Violence Prevention Charter.

While the Social Network Analysis characterized the relational structure of the PHABC network and identified the organizational conditions supporting dissemination, it did not explain the contextual reasoning underlying participants’ decisions to engage with or decline the Violence Prevention Charter. These questions were explored through the realist-informed qualitative inquiry presented in the following section.

### 3.3 Realist-informed findings

#### 3.3.1 Focus groups and coding

Focus group members were drawn from the membership of the Public Health Association of BC. Three different focus group sessions were conducted in order to reach the designated number of participants (N = 12). The number of participants in the focus groups was limited to 12 for two reasons. Twelve represents 24% of the core membership and 8% of the active membership and as such would not detract for later research with the same population and would still meet the demand for theoretical sufficiency vs. saturation (Vasileiou et al., 2018). Two, the minimum sample size for data saturation in qualitative studies indicated by Clark and Braun (2013) was 12. In fact, saturation was achieved with 12 participants as coded results demonstrated consistency in response to thematic analysis.

The first session had four participants, the second session had three participants with one person joining from session 1 to complete the Mentimeter survey, the final session had three participants. Three participants chose to provide their feedback online via the Mentimeter survey. Questions in the Mentimeter survey were designed to solicit opinion and experience from focus group respondents and were the basis for a deeper discussion of context and decision making with regard to the VPC. Given the supportive and pro-social context of PHABC, it was anticipated that members would be motivated to participate in the online Violence Prevention Charter process. Nevertheless, it was necessary to clarify what the motivating mechanism was for participation and why some members chose not to support the VPC.

Both coders reviewed the each other’s findings and, in this process, validated assignment of content within the secondary frame. This process was straightforward and there were no disagreements regarding assignments to the frames. The coders worked independently and those results were then assessed collectively with discussion until unanimous agreement was reached. Figure 4 shows the integrated results of the coding process where context and sentiment were the organized focus of the assessment.

**Figure 4:**
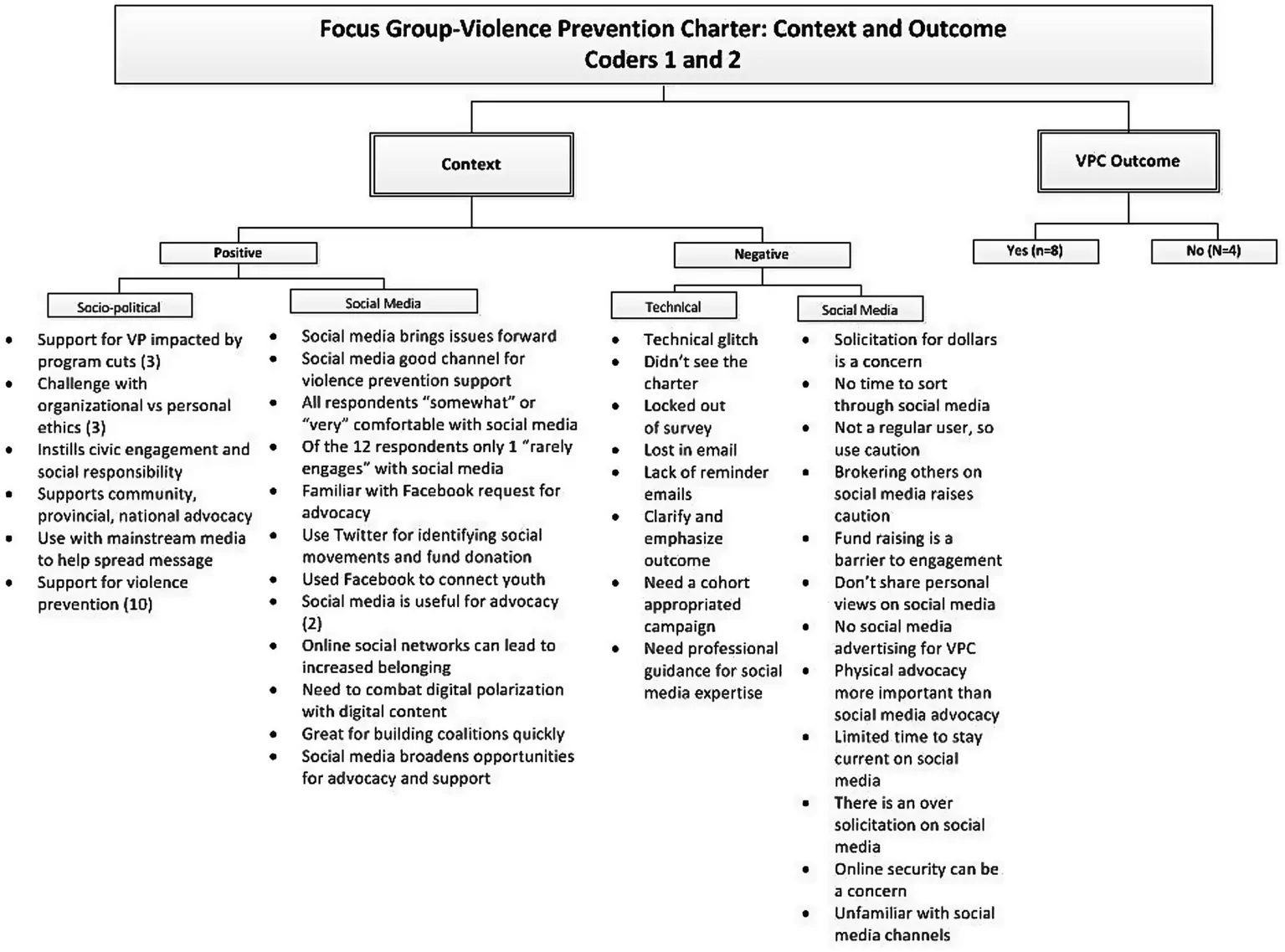
Focus group-violence prevention charter: context and outcomes – coders 1 and 2. From focus group teleconference meetings, August and December 2019.

The realist-informed inquiry explored the contextual conditions and generative mechanisms underlying participant decisions to engage with the Violence Prevention Charter. Focus groups were undertaken to examine the reasoning behind decisions to sign or decline the Charter and to identify the Context–Mechanism–Outcome (CMO) configurations associated with voluntary participation. Findings from the qualitative inquiry informed development of the subsequent Social Network Analysis survey, providing an opportunity to examine whether the identified mechanisms were evident across the broader PHABC network. This sequential explanatory approach is consistent with realist inquiry, which seeks to explain how interventions work by examining how participants reason and respond to program resources within particular contexts (31, 32). More recent realist scholarship has extended this perspective by emphasizing that mechanisms are not fixed properties of interventions but emerge through the interaction between program resources, participant reasoning, and context, particularly within complex public health systems (33, 34).

#### 3.3.2 Context–mechanism–outcome configurations identified through realist-informed qualitative analysis

The focus-group analysis identified three principal generative mechanisms influencing engagement with the Violence Prevention Charter: creation of social good, trust in the source, and influence of colleagues and peers. These mechanisms were identified independently by both coders and were subsequently reconciled through the coding process. Within the social good theme, participants connected their decision-making to the value of violence prevention, advocacy, and a broader moral commitment to public health. One participant described the desired outcome simply as “safety, security and peace for all.”

Trust in the source was similarly prominent. Participants distinguished between online requests originating from organizations they knew and trusted and those from unfamiliar sources. PHABC was described as an “evidence based source,” and its established credibility provided an important context for engagement with the Charter. This reasoning was expressed more fully by one participant:

“It is important for me to have a trusted and known organization that is focusing around the online advocacy.”

The participant explained that although they sometimes engaged in online advocacy, requests from organizations they only vaguely knew generated uncertainty about the purpose and direction of the advocacy. Trust therefore operated through participants’ assessment of the credibility of the organization communicating the request rather than through exposure to the online message alone.

The third mechanism was influence from colleagues and peers. Participants referred to PHABC colleagues, organizational relationships, conference participation, and prior exposure to violence-prevention activities when explaining their decision to support the Charter. One participant identified a violence-prevention workshop at a PHABC conference as contributing to their decision to sign, while others reported signing following encouragement from a PHABC colleague. The coding therefore situated peer influence within an existing professional social network rather than as a generalized effect of social media.

The CMO analysis also identified contextual conditions that could inhibit engagement even where participants were supportive of violence prevention. Participants who had not signed described overflowing inboxes, competing demands on their time, the need for reminders, unfamiliar online solicitations, and concerns about subsequent requests for donations. One participant described this constraint directly:

“I think it’s a time issue, by the time I go through Facebook and email and all messages regarding my friend who advocate for this and that and I feel I’ve used my time that way, so it’s a time issue for me.”

This response is important because non-adoption did not necessarily indicate disagreement with the Charter or its violence-prevention objectives. Rather, practical conditions could prevent an otherwise supportive individual from acting on the invitation. Participants indicated that barriers such as time, competing online communication and lack of follow-up could potentially be overcome through reminders or communication from a trusted organization.

Participants also differentiated online advocacy from other forms of public-health action. One participant described using social media selectively for issues operating at a broader scale:

“I use social media very erratically; I tend to do the social media and a group of things and I was worried I was getting lazy and not getting involved in the more local activist things and so I am using social media for issues I care about on a national or provincial scale.”

This illustrates the agency involved in participants’ decisions about whether and how to engage digitally. Although all focus-group participants reported being at least somewhat comfortable with social media, they did not describe themselves as regular users, reinforcing the distinction between technological capability and a decision to participate in online advocacy.

The resulting CMO configuration therefore suggests that voluntary adoption occurred when the Charter’s violence-prevention purpose aligned with participants’ existing public-health values, the request was communicated through a credible and trusted source, and professional relationships reinforced its relevance. Conversely, competing demands, uncertainty regarding online requests, and characteristics of the digital environment could interrupt engagement despite underlying support for violence prevention. The principal outcomes were decisions to adopt or decline the Charter and subsequent willingness to share or advocate for its principles. These qualitative findings subsequently informed the Social Network Analysis questions examining trust and professional influence across the broader PHABC network.

Participants consistently identified creation of social good, trust in shared information, influence of colleagues and peers, and professional commitment to violence prevention as the principal generative mechanisms influencing engagement with the Violence Prevention Charter.

These mechanisms were activated within contexts characterized by established professional relationships, organizational credibility, alignment with shared public health values, and confidence in the sponsoring organizations. Consistent with realist methodology, these mechanisms reflected participants’ reasoning in response to contextual conditions rather than attributes of the intervention itself (31, 35). This interpretation aligns with Shaw et al. (34), who argue that mechanisms should be understood as the reasoning and responses triggered when participants interact with program resources under particular contextual conditions. The mechanisms identified in the present study reflect this conceptualization, demonstrating that trust, professional influence, commitment to violence prevention, and the creation of social good emerged through participants’ interpretation of the intervention rather than from the intervention itself.

The contextual conditions influencing these mechanisms included participants’ familiarity with the Violence Prevention Charter, existing professional relationships, organizational roles, and confidence in the credibility of the information being communicated. Within these contexts, trust and professional influence acted as generative mechanisms supporting voluntary engagement, while competing priorities, uncertainty regarding the initiative, and limited familiarity with the Charter reduced the likelihood of participation. These findings demonstrate that engagement was shaped by participants’ reasoning within their professional context rather than by exposure to online communications alone. Similar findings have been reported in contemporary realist evaluations of complex health interventions, where differences in implementation are explained through variations in stakeholder reasoning and local context rather than through differences in the intervention itself (36). Jagosh (33) likewise argues that understanding how mechanisms are activated across diverse contexts provides a more meaningful explanation of program outcomes than describing implementation processes alone.

The principal outcomes included decisions to adopt or decline the Violence Prevention Charter, willingness to share Charter information with colleagues, and varying levels of advocacy and organizational engagement. Importantly, the subsequent Social Network Analysis demonstrated high levels of trust and professional influence across the PHABC network, providing empirical support for the mechanisms identified through the focus groups. Together, the qualitative and quantitative findings demonstrated that voluntary adoption resulted from the interaction between contextual conditions and generative mechanisms rather than from the intervention alone.

Taken together, the realist-informed findings provided the explanatory link between the observed patterns of diffusion and organizational network structure. While diffusion modeling demonstrated the timing and trajectory of dissemination, and Social Network Analysis characterized the professional relationships through which information travelled, the realist-informed inquiry explained the reasoning underlying participants’ decisions to engage with the Violence Prevention Charter. These complementary findings provide the basis for the integrated interpretation presented in the Discussion.

## 4 Discussion

### 4.1 Integrated interpretation of the findings

This study integrated web analytics, Bass diffusion modeling, Social Network Analysis (SNA), and a realist-informed examination of Context–Mechanism–Outcome (CMO) configurations to investigate dissemination and adoption decision-making associated with a principles-based digital public health intervention. Rather than considering these findings independently, the sequential mixed-methods design generated complementary temporal, relational, and explanatory evidence that provided a more comprehensive understanding of dissemination than any individual analytical approach could achieve.

The findings demonstrated measurable engagement following the public release of the Violence Prevention Charter, followed by accelerated diffusion after implementation of a targeted promotional intervention. Consistent with Rogers’ Diffusion of Innovations theory, dissemination increased awareness and accelerated adoption through communication within a social system (2). Similarly, the Bass Diffusion Model (1) characterized the observed adoption pattern, demonstrating how innovation adoption progressed beyond initial uptake as dissemination activities expanded across the network. However, the subsequent phases of the investigation demonstrated that dissemination alone did not explain participant engagement. Social Network Analysis characterized the professional relationships through which information circulated within the PHABC network and participating partner organizations, while the realist-informed inquiry explained how participants interpreted the Charter within their own organizational contexts and why they chose to engage. Together, these complementary findings demonstrate that digital dissemination is best understood not as a single communication event but as a dynamic process shaped by interactions among communication, professional relationships, organizational context, and participant reasoning.

An important practical observation emerging from the investigation is that promotion requires promotion. Although the Charter generated measurable engagement following its public release, purposeful dissemination substantially accelerated adoption. This finding reinforces Rogers' (2) observation that communication is fundamental to innovation diffusion while extending it by demonstrating that communication alone is insufficient to explain adoption within contemporary digital public health environments. Dissemination established opportunities for engagement, but adoption reflected decisions made by individuals and organizations within existing professional relationships, organizational contexts, and shared public health values.

Importantly, no single method fully explained the dissemination process. Web analytics and diffusion modeling identified when engagement occurred and how adoption evolved over time. Social Network Analysis examined through whom information and influence circulated, while the realist-informed inquiry explained why participants chose to engage under differing contextual conditions. Considered together, these complementary methods provided a richer understanding of dissemination and adoption decision-making than any individual analytical approach could achieve. This integrated interpretation forms the foundation for the subsequent discussion of digital public health ecosystems, Complex Adaptive Systems, and the methodological contribution of the sequential mixed-methods framework.

### 4.2 Digital ecosystems as settings for health promotion

The findings suggest that dissemination of the Violence Prevention Charter occurred within a broader digital public health ecosystem rather than through isolated communication channels. Information moved across multiple organizational and professional networks, participants interpreted shared principles according to their own organizational contexts, and decisions to engage reflected interactions among communication processes, professional relationships, organizational priorities, and individual reasoning. Rather than functioning solely as a communication platform, the digital environment served as a contemporary setting within which health promotion activities, collaboration, and organizational decision-making occurred.

This perspective extends the settings-based approach established in the Ottawa Charter for Health Promotion, which recognizes that health is created and experienced within the settings of everyday life. Increasingly, these settings include digitally connected environments where information, relationships, and social interaction influence health-related decision-making across multiple levels of the socio-ecological model. Within this investigation, the digital ecosystem provided an environment in which public health organizations exchanged information, interpreted shared principles, strengthened professional relationships, and voluntarily coordinated action around violence prevention. In this way, the digital environment functioned not simply as a dissemination tool, but as a setting that supported health promotion through communication, collaboration, and shared learning.

Recent scholarship further strengthens this interpretation. Kickbusch and Holly (37) argue that digital environments increasingly shape the social, political, commercial, and informational conditions that influence health and wellbeing, positioning digital ecosystems as important determinants of health rather than simply channels for communication. From this perspective, digital technologies influence how health information is created, shared, interpreted, and acted upon while simultaneously shaping the relationships and organizational networks through which public health practice occurs. Similarly, emerging scholarship in digital public health recognizes that digital ecosystems comprise interconnected social, organizational, and technological environments in which multiple actors interact, adapt, and exchange information in ways that influence public health action. The findings of the present study are consistent with this perspective, demonstrating that dissemination was influenced not only by communication reach but also by trust, organizational credibility, professional relationships, and contextual interpretation.

Importantly, these findings do not suggest that digital technologies themselves produce collaboration or behavior change. Rather, they demonstrate that digital ecosystems create opportunities for interaction within which existing professional relationships, organizational contexts, and participant reasoning influence dissemination and voluntary adoption. This distinction is important because it recognizes that technologies support—but do not determine—public health action. Instead, meaningful engagement emerged through interactions among communication processes, trusted professional relationships, organizational context, and shared public health values.

The principles-based design of the Violence Prevention Charter further illustrates this relationship. Rather than prescribing standardized implementation activities, the Charter encouraged organizations to interpret shared public health principles according to their own priorities, capacities, and community contexts. This flexibility enabled organizations to exercise professional judgement while remaining connected through a common set of violence prevention principles. Consistent with health promotion theory, the intervention therefore supported locally appropriate action rather than uniform implementation, reinforcing the importance of enabling environments that encourage participation, collaboration, and adaptation across diverse public health settings.

Taken together, these findings suggest that digital ecosystems should be understood as contemporary settings for health promotion in which dissemination, collaboration, and decision-making emerge through dynamic interactions among communication technologies, professional relationships, organizational contexts, and individual agency. Extending established health promotion theory into digitally connected environments provides a stronger conceptual foundation for understanding principles-based public health interventions and reinforces the value of investigating digital ecosystems as adaptive settings within which health promotion practice increasingly occurs.

### 4.3 Independent decision-making within trusted social networks

The integration of findings across the sequential mixed-methods investigation provides important insight into how participants engaged with the Violence Prevention Charter. Temporal analyses demonstrated when engagement occurred and diffusion accelerated. Bass diffusion modeling characterized the pattern of adoption over time. Social Network Analysis described the relational pathways through which information and shared principles circulated within the PHABC network and participating partner organizations, while the realist-informed inquiry explained how participants interpreted the Charter within their own organizational contexts and why they chose to engage. Together, these complementary methods demonstrate that dissemination cannot be fully understood without considering both the relationships through which information travels and the contextual reasoning that influences adoption decisions.

Across these complementary analyses, one finding remained remarkably consistent: engagement occurred through trusted professional relationships rather than through communication exposure alone. The Social Network Analysis demonstrated high levels of trust, collaboration, and connectivity among organizations within the PHABC network, while the realist-informed inquiry identified confidence in participating organizations, credibility of the Charter, commitment to violence prevention, opportunities for collaboration, and alignment with shared public health values as mechanisms influencing participant engagement. Collectively, these findings suggest that dissemination reflected more than the transmission of information; it represented a process of professional interpretation occurring within established relationships characterized by trust and shared purpose.

Contemporary realist scholarship provides additional insight into this interpretation. Shaw et al. (34) emphasize that implementation outcomes emerge through the interaction between contextual conditions and participant reasoning rather than from intervention characteristics alone. Similarly, Jagosh (33) argues that realist inquiry is particularly valuable for understanding complex interventions because it seeks to explain how and why outcomes vary across different contexts rather than determining whether an intervention simply works. These perspectives closely align with the present findings, where participant engagement was influenced by how organizations interpreted the Violence Prevention Charter within their own mandates, priorities, capacities, and existing partnerships rather than by the Charter itself.

This interpretation is further supported by Flynn et al. (36), who demonstrated that variation in implementation frequently reflects differences in local organizational contexts and the mechanisms those contexts activate. Consistent with this perspective, the present investigation found that organizations were not passive recipients of information but active decision-makers who assessed the relevance of the Charter within their own environments. Shared public health principles provided a common foundation, while local organizational contexts influenced how those principles were interpreted and applied. The observed variation in engagement therefore reflects the adaptive nature of public health practice rather than inconsistency in the intervention itself.

Importantly, these findings should not be interpreted as suggesting that trusted relationships alone determine dissemination. Rather, trust functioned as one component within a broader network of interacting influences that included communication, organizational credibility, participant reasoning, and contextual opportunity. Decisions to endorse, promote, or implement the Charter reflected informed professional judgement supported by existing relationships and shared public health values. This interpretation is consistent with both diffusion theory and realist evaluation, while reinforcing the importance of understanding dissemination as a socially mediated and contextually situated process.

The findings also extend understanding of professional public health networks within digital environments. The Social Network Analysis demonstrated that relationships within the PHABC network provided the relational architecture through which information and influence circulated, while the realist-informed inquiry explained why those relationships contributed to participant engagement. Viewed together, these complementary findings suggest that effective digital dissemination depends not simply on expanding communication reach but on fostering trusted relationships that support professional dialogue, collaborative learning, and independent decision-making. Public health organizations may therefore achieve greater impact by strengthening existing networks and enabling meaningful engagement than by relying solely on increasingly sophisticated communication technologies.

### 4.4 Understanding diffusion through a complex adaptive systems perspective

The integration of web analytics, Bass diffusion modeling, Social Network Analysis, and realist-informed inquiry generated complementary evidence describing different dimensions of the same dissemination process. No individual method was intended to explain the complexity of intervention diffusion independently. Rather, each contributed a distinct perspective that, when considered collectively, provided a more comprehensive understanding of dissemination and adoption decision-making within the PHABC network and participating partner organizations.

A Complex Adaptive Systems (CAS) perspective provides the most coherent framework for interpreting these integrated findings. Rather than conceptualizing dissemination as a linear communication process, CAS recognizes public health systems as dynamic networks of interacting individuals, organizations, and environments whose behaviors continually influence one another through feedback, adaptation, learning, and self-organization. From this perspective, dissemination is understood as an emergent process arising from multiple interacting relationships rather than a simple consequence of communication exposure alone.

Contemporary complexity scholarship reinforces this interpretation. Greenhalgh and Papoutsi (13) argue that health interventions should not be viewed as acting upon static organizational systems but as becoming embedded within adaptive networks of relationships that continually evolve through interaction, contextual adaptation, and learning. This perspective recognizes that interventions both influence and are influenced by the systems in which they operate. The findings of the present study are consistent with this interpretation. Diffusion modeling described the temporal dynamics of dissemination, Social Network Analysis characterized the relational architecture through which information and influence circulated, and the realist-informed inquiry explained how contextual conditions and participant reasoning shaped engagement. Together, these complementary findings illustrate how multiple interacting processes contributed to dissemination within a digital public health ecosystem.

Importantly, this study does not claim to demonstrate complexity empirically or to provide direct evidence of emergence or system transformation. Rather, the observed patterns are consistent with a Complex Adaptive Systems perspective that offers a coherent explanatory framework for interpreting the converging evidence generated through the sequential mixed-methods investigation. This distinction is important because the study was designed to investigate dissemination and adoption decision-making, not to measure the behavior of the system itself. The value of the CAS perspective therefore lies in its ability to explain how communication processes, professional relationships, organizational context, and participant reasoning interacted to influence dissemination across the network.

The findings also reinforce the importance of agency within complex systems. Organizations did not respond uniformly to the Violence Prevention Charter, nor were they expected to. Instead, participants interpreted shared violence prevention principles according to their own organizational mandates, priorities, partnerships, and community contexts. These locally informed decisions contributed to the overall dissemination process while preserving organizational autonomy. Such adaptive responses are consistent with principles of health promotion and complexity science, where locally situated decision-making is understood to strengthen rather than diminish system resilience and innovation.

Viewed collectively, the findings suggest that dissemination of principles-based public health interventions is best understood as an adaptive process operating within a complex digital ecosystem. Communication created opportunities for engagement, professional networks facilitated the circulation of information and influence, organizational contexts shaped interpretation, and participant reasoning informed adoption decisions. The sequential integration of temporal, relational, and explanatory evidence therefore provides a richer understanding of dissemination than any individual methodological approach alone and demonstrates the value of interpreting digital public health interventions through a Complex Adaptive Systems perspective.

### 4.5 Methodological contribution

The principal methodological contribution of this investigation lies not in the individual application of web analytics, Bass diffusion modeling, Social Network Analysis (SNA), or realist-informed inquiry, but in their deliberate sequential integration. Each method examined a complementary dimension of dissemination and adoption decision-making. Web analytics and diffusion modeling characterized the temporal dynamics of dissemination, Social Network Analysis examined the relational architecture through which information and influence circulated within the PHABC network and participating partner organizations, and the realist-informed inquiry explained the contextual conditions and generative mechanisms that influenced participant engagement. Considered together, these methods generated complementary temporal, relational, and explanatory evidence describing the same adaptive phenomenon.

The sequential mixed-methods design extends existing approaches to evaluating digital public health interventions by recognizing that no single methodological perspective adequately explains dissemination within complex organizational environments. Digital public health interventions increasingly operate within systems characterized by multiple interacting actors, decentralized decision-making, evolving communication pathways, and diverse organizational contexts. Investigating these interventions therefore requires approaches capable of examining not only dissemination outcomes but also the relationships, contextual influences, and decision-making processes through which adoption occurs.

From a public health informatics perspective, the findings reinforce the value of integrating complementary analytical methods to investigate digital public health interventions. Public health informatics has increasingly emphasized understanding the interactions among people, information, technology, and organizational systems to improve public health practice and decision-making (38–40). While web analytics, network analysis, and implementation evaluation each provide valuable but distinct forms of evidence, their deliberate integration offers a more comprehensive understanding of dissemination than any individual method alone. The present investigation demonstrates how quantitative measures of dissemination can be strengthened through complementary relational and explanatory analyses, providing a richer understanding of how digital public health interventions are communicated, interpreted, and adopted within professional public health networks.

The methodological contribution also extends contemporary implementation science by demonstrating the value of combining diffusion theory, network science, and realist-informed inquiry within a single investigative sequence. Rather than privileging one methodological perspective, the sequential design acknowledges that complex public health interventions generate multiple forms of evidence, each contributing unique insight into dissemination and adoption decision-making. This integrated approach is consistent with calls for more pluralistic methodological frameworks capable of investigating complexity within health systems while remaining grounded in empirical observation (13, 33).

The principal contribution of this investigation therefore lies in demonstrating a sequential mixed-methods framework capable of integrating temporal, relational, and explanatory evidence within a single coherent analysis. Beyond the Violence Prevention Charter, this framework offers a transferable approach for investigating dissemination and adoption decision-making associated with principles-based digital public health interventions operating within complex adaptive systems. More broadly, it contributes to the methodological foundations of digital public health, public health informatics, and implementation science by illustrating how complementary analytical approaches can be integrated to investigate increasingly complex public health challenges.

### 4.6 Implications for public health practice

Several practical implications emerge from this investigation for the design and implementation of principles-based digital public health interventions.

First, the findings reinforce that promotion requires promotion. Although the Violence Prevention Charter generated measurable engagement following its public release, purposeful dissemination substantially accelerated adoption. Simply making digital resources publicly available is unlikely to maximize awareness or engagement without deliberate communication strategies that promote visibility across professional networks. For public health organizations, dissemination should therefore be viewed as an active process requiring ongoing communication rather than a single publication event.

Second, the findings highlight the importance of trusted professional relationships in supporting dissemination and adoption. Communication reach alone did not explain participant engagement. Rather, organizations chose to engage because the Charter aligned with shared public health values, was introduced through credible professional relationships, and could be interpreted within their own organizational mandates and community contexts. These findings suggest that dissemination strategies are likely to be more effective when they build upon existing networks of trust and collaboration than when they rely exclusively on increasingly sophisticated communication technologies or broad messaging campaigns.

Third, the findings demonstrate the value of principles-based public health interventions for addressing complex public health issues. Rather than prescribing standardized implementation activities, the Violence Prevention Charter encouraged organizations to interpret and apply shared violence prevention principles according to their own priorities, capacities, and community needs. This flexibility supported locally appropriate responses while maintaining alignment with common public health objectives. Such an approach reflects established principles of health promotion by enabling organizations to exercise professional judgement while contributing to broader collective goals.

More broadly, the findings suggest that digital public health interventions should be designed with equal attention to communication, relationships, organizational context, and participant reasoning. Effective dissemination depends not only on communicating information but also on creating opportunities for meaningful interaction, collaboration, and shared learning within trusted professional networks. Understanding digital ecosystems as contemporary settings for health promotion therefore encourages a shift from viewing technology as the intervention itself to recognizing technology as part of a broader environment that supports collaboration, knowledge exchange, and adaptive public health practice.

Finally, the sequential mixed-methods framework demonstrated in this investigation offers practical value beyond evaluation alone. By integrating temporal, relational, and explanatory evidence, the framework provides public health practitioners and researchers with a structured approach for understanding not only whether dissemination occurs, but how, through whom, and why adoption decisions are made. This broader understanding may support the design, implementation, and evaluation of future digital public health initiatives operating within increasingly complex organizational environments.

### 4.7 Limitations and future research

The findings should be interpreted within the context and purpose of this investigation. This study examined dissemination and adoption decision-making associated with a principles-based violence prevention intervention within the PHABC network and participating partner organizations. As a sequential mixed-methods case study, the objective was not to establish causal relationships or produce findings intended for statistical generalization. Rather, the investigation sought to develop a richer understanding of dissemination by integrating complementary temporal, relational, and explanatory forms of evidence within a single analytical framework. The intention was to generate an empirically grounded explanation of dissemination and adoption decision-making that may inform understanding of similar public health interventions while recognizing that the transferability of these findings depends upon the characteristics of the settings in which they are applied.

Several methodological considerations should therefore be acknowledged. First, the investigation examined a single principles-based intervention operating within one bounded digital public health ecosystem. Although the findings provide detailed insight into dissemination and adoption decision-making within this context, other public health priorities, organizational structures, digital environments, and professional networks may demonstrate different patterns of dissemination and engagement. Rather than seeking universal explanation, the study contributes contextually grounded knowledge that may be transferable to other settings where similar conditions, relationships, and implementation approaches exist.

Second, each methodological component contributed insight into a distinct dimension of dissemination while also possessing inherent limitations when considered independently. Web analytics and diffusion modeling characterized patterns of engagement over time but could not explain why individuals or organizations chose to engage. Social Network Analysis described the relational architecture of the PHABC network and participating partner organizations but did not determine how relationships influenced participant reasoning or organizational decision-making. Conversely, the realist-informed inquiry provided explanatory insight into contextual influences and generative mechanisms but was not intended to quantify the relative contribution of individual mechanisms or predict dissemination outcomes. The deliberate sequential integration of these complementary methods was therefore central to the investigative design, allowing the strengths of one approach to inform, extend, and explain the findings of the others.

Third, although Complex Adaptive Systems provided a coherent explanatory framework for interpreting the integrated findings, the investigation was not designed to measure system behavior directly or demonstrate emergence empirically. Rather, the observed dissemination patterns were interpreted as being consistent with a Complex Adaptive Systems perspective. This distinction is important because it positions complexity as a theoretical framework through which complementary evidence can be understood rather than as an outcome measured by the investigation itself. Maintaining this distinction strengthens the interpretation by ensuring that conclusions remain aligned with the scope of the empirical evidence.

The principles-based nature of the Violence Prevention Charter should also be considered when interpreting the findings. Unlike interventions that prescribe standardized implementation activities, the Charter encouraged organizations to interpret and apply shared principles according to their own priorities, capacities, and community contexts. Variation in organizational responses was therefore anticipated and reflects an important characteristic of principles-based health promotion interventions rather than inconsistency in implementation. The findings should consequently be interpreted as demonstrating how organizations exercised independent professional judgement within shared public health principles rather than evaluating adherence to a prescribed implementation model.

These methodological boundaries also identify important opportunities for future research. Application of the sequential mixed-methods framework across different public health interventions, organizational settings, and digital ecosystems would strengthen understanding of its transferability and methodological utility. Longitudinal investigations could examine how dissemination, collaboration, and organizational learning evolve over time, while comparative studies may explore how differing network structures, governance arrangements, and organizational contexts influence adoption decision-making. Additional research examining digital ecosystems as contemporary settings for health promotion would further advance understanding of how communication technologies, professional relationships, adaptive organizational systems, and contextual influences interact to shape public health practice.

Ultimately, the methodological boundaries of the individual analytical approaches reinforce one of the principal contributions of this investigation. No single method fully explained dissemination and adoption decision-making within this complex public health environment. By deliberately integrating temporal, relational, and explanatory forms of evidence, the sequential mixed-methods design provided a more comprehensive understanding of dissemination than any individual analytical approach could have achieved independently. This integrated investigative framework therefore offers a transferable approach for future research seeking to understand the complexity of digital public health interventions operating within adaptive organizational systems.

## 5 Conclusion

This investigation contributes a sequential mixed-methods framework for understanding dissemination and adoption decision-making associated with principles-based digital public health interventions. By integrating web analytics, Bass diffusion modeling, Social Network Analysis, and a realist-informed examination of Context–Mechanism–Outcome (CMO) configurations, the study generated complementary temporal, relational, and explanatory evidence describing how dissemination occurred, through whom information and influence travelled, and why individuals and organizations chose to engage.

Interpreted through a Complex Adaptive Systems perspective, the findings suggest that dissemination within digital public health ecosystems is shaped not only by communication processes but also by interactions among professional relationships, organizational context, participant reasoning, and shared public health values. The investigation demonstrates that purposeful dissemination accelerated adoption, while trusted professional networks and locally situated decision-making influenced how organizations interpreted and applied shared violence prevention principles. These findings reinforce the importance of understanding dissemination as an adaptive process operating within contemporary digital ecosystems rather than as a linear sequence of communication events.

The study also advances the conceptualization of digital ecosystems as contemporary settings for health promotion. Rather than functioning solely as channels for information exchange, digital environments provided settings in which organizations collaborated, interpreted shared principles, and independently determined appropriate courses of action within their own communities and organizational contexts. Understanding digital ecosystems in this way extends established health promotion theory while providing a stronger conceptual foundation for investigating digital public health interventions.

The principal contribution of this research lies not in the individual application of diffusion modeling, Social Network Analysis, or realist-informed inquiry, but in demonstrating how these complementary methods can be deliberately integrated within a sequential mixed-methods investigative framework. By combining temporal, relational, and explanatory forms of evidence, the framework provides a richer understanding of dissemination and adoption decision-making than any individual analytical approach alone. Beyond the Violence Prevention Charter, this investigative framework offers a transferable approach for future research examining principles-based interventions operating within complex public health systems.

As digital technologies become increasingly embedded within public health practice, understanding how information, relationships, organizational context, and participant reasoning interact to influence dissemination will become increasingly important. This investigation contributes to the growing fields of digital public health, public health informatics, implementation science, and health promotion by providing an empirically grounded framework for investigating dissemination within contemporary digital public health ecosystems.

## Data Availability

The original contributions presented in the study are included in the article/supplementary material, further inquiries can be directed to the corresponding author/s.
